# Capsid amino acids at positions 247 and 270 are involved in the virulence of betanodaviruses to European sea bass

**DOI:** 10.1038/s41598-019-50622-1

**Published:** 2019-10-01

**Authors:** Patricia Moreno, Sandra Souto, Rocio Leiva-Rebollo, Juan J. Borrego, Isabel Bandín, M. Carmen Alonso

**Affiliations:** 1 Universidad de Málaga, Departamento de Microbiología, Málaga, Spain; 2 Departamento de Microbiología y Parasitología, Instituto de Acuicultura, Universidad de Santiago de Compostela, Santiago de Compostela, Spain

**Keywords:** Infectious diseases, Viral pathogenesis

## Abstract

European sea bass (*Dicentrarchus labrax*) is severely affected by nervous necrosis disease, caused by nervous necrosis virus (NNV). Two out of the four genotypes of this virus (red-spotted grouper nervous necrosis virus, RGNNV; and striped jack nervous necrosis virus, SJNNV) have been detected in sea bass, although showing different levels of virulence to this fish species. Thus, sea bass is highly susceptible to RGNNV, whereas outbreaks caused by SJNNV have not been reported in this fish species. The role of the capsid protein (Cp) amino acids 247 and 270 in the virulence of a RGNNV isolate to sea bass has been evaluated by the generation of recombinant RGNNV viruses harbouring SJNNV-type amino acids in the above mentioned positions (Mut247Dl965, Mut270Dl965 and Mut247 + 270Dl965). Viral *in vitro* and *in vivo* replication, virus virulence and fish immune response triggered by these viruses have been analysed. Mutated viruses replicated on E-11 cells, although showing some differences compared to the wild type virus, suggesting that the mutations can affect the viral cell recognition and entry. *In vivo*, fish mortality caused by mutated viruses was 75% lower, and viral replication in sea bass brain was altered compared to non-mutated virus. Regarding sea bass immune response, mutated viruses triggered a lower induction of IFN I system and inflammatory response-related genes. Furthermore, mutations caused changes in viral serological properties (especially the mutation in amino acid 270), inducing higher seroconversion and changing antigen recognition.

## Introduction

Nervous necrosis virus (NNV), *Nodaviridae* family, *Betanodavirus* genus, is the causative agent of viral encephalopathy and retinopathy (VER), otherwise known as viral nervous necrosis (VNN), a serious disease affecting brain and retina of a wide range of fish species worldwide. NNV genome is composed of two single-stranded positive-sense genomic segments, RNA1 and RNA2, encoding the viral polymerase and the capsid protein (Cp), respectively^1^. In addition, a subgenomic RNA (RNA3), which codifies two non-structural proteins (B1 and B2)^2,3^, is transcribed from the RNA1 3′ end. On the basis of the phylogenetic analysis of a variable region within the RNA2 segment, betanodaviruses are classified into four genotypes: striped jack nervous necrosis virus (SJNNV), tiger puffer nervous necrosis virus (TPNNV), barfin flounder nervous necrosis virus (BFNNV), and red-spotted grouper nervous necrosis virus (RGNNV)^4^. Moreover, reassortant isolates harbouring RGNNV and SJNNV genomic segments (in both RNA1/RNA2 combinations: SJNNV/RGNNV and RGNNV/SJNNV) have been reported^5–7^.

The virulence of a virus depends on several factors related to the pathogen, host, and host-pathogen interaction. Regarding the pathogen, the viral virulence is determined by multiple factors, including host-cell recognition and entry, immune system antagonism mechanisms, and viral replication efficiency. In this concern, the Cp C-terminus region has been described as an important determinant of betanodavirus virulence and host specificity^8–10^. In particular, two amino acids located in the Cp protruding domain (P-domain) have been suggested as potential betanodavirus virulence determinants^11^.

Several host factors, such as age, rearing conditions, feeding, and immunological state, may influence the disease severity. In this regard, it is important to highlight the role of fish innate immune system as the first barrier against virus infections, being especially relevant the interferon I system (IFN I), which promotes an antiviral state by inducing the transcription of interferon-stimulated genes (ISGs)^12^, and the inflammatory response, which seems to be especially important to control nodavirus infections.

European sea bass (*Dicentrarchus labrax*) is highly susceptible to RGNNV infections^13–17^, whereas the mortality recorded in this fish species after SJNNV infection is very low^16,18^. Moreover, natural outbreaks caused by SJNNV have not been reported in this fish species to date. Interestingly, a previous study has reported a RGNNV/SJNNV reassortant strain, isolated from Senegalese sole (*Solea senegalensis*), which, despite of displaying a SJNNV-type Cp, caused 33% mortality in experimentally infected sea bass^11^. The virulence of this reassortant strain to European sea bass has been proposed to be associated with the presence of RGNNV-type amino acids at positions 247 and 270 of the SJNNV Cp sequence (serine in both positions)^11^. In addition, the Cp sequence analysis of several RGNNV/SJNNV-type reassortants naturally pathogenic to gilthead seabream (*Sparus aurata*) showed also the presence of RGNNV-type amino acids at the same positions^7^.

Therefore, the aim of the present study has been to evaluate the role of amino acids 247 and 270 in the Cp sequence as betanodavirus virulence determinants to European sea bass. To fulfil this aim, a RGNNV recombinant strain, generated by reverse genetics, has been subjected to site-directed mutagenesis at both positions, which changed the above mentioned amino acids to those present in a SJNNV-type Cp. The effect of these mutations on viral replication, virulence to sea bass, and host immune response has been analysed.

## Results

### Recovery of *r*Dl965 and mutated viruses

Reverse genetics methodology allowed to obtain recombinant Dl965-derived viral particles (*r*DI965) from viral cDNA with identical sequence to the wild type virus (*wt*Dl965), or with mutations in the RNA2 segment at positions 247 (Mut247Dl965), 270 (Mut270Dl965) and both positions (Mut247 + 270Dl965). As a result of the mutations, amino acids at positions 247 (serine) and 270 (serine) were replaced by those amino acids at the same positions in SJNNV isolates (alanine and asparagine, respectively). Mutations were confirmed by PCR, obtaining a single band with the expected size (568 bp), followed by sequencing (data non-shown). The infective viral particles obtained after BSRT7/5 cell line transfection were propagated on E-11 cells, recording typical cytopathic effects (CPE) after the first cell inoculation (P0) for *r*Dl965-inoculated cells, whereas one blind passage (P1) was required for cells inoculated with mutant viruses. The titres of the viral stocks used for the subsequent virulence analyses (P2 on E-11 cells) were 7.1 × 10^7^, 1.3 × 10^8^, 7.1 × 10^7^, and 2.3 × 10^7^ TCID_50_/ml, for *r*Dl968, Mut247Dl965, Mut270Dl965, and Mut247 + 270Dl965, respectively.

### Replication kinetics of recombinant viruses

**Figure 1:**
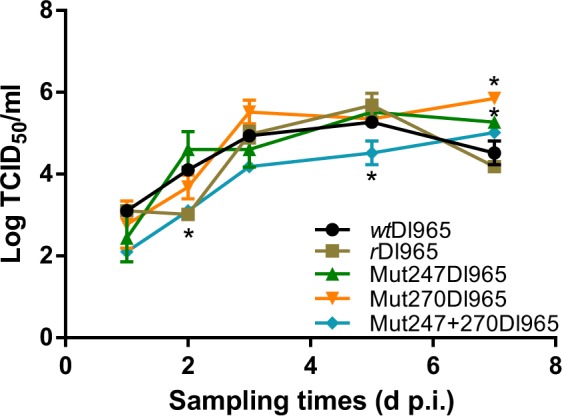
Viral replication on the E-11 cell line inoculated at 0.1 multiplicity of infection (MOI). The one-way ANOVA with Bonferroni’s multiple comparisons test were the statistical analyses carried out. Asterisk represents significant differences compared to *wt*Dl965 within each sampling time (p < 0.05). Results are mean ± standard deviation (s.d.) (n = 3).

Replication of the different recombinant viruses was analysed on E-11 cells and compared to *wt*Dl965 replication. All viruses replicated on E-11 cells, with an important increase of the viral titre from 24 h post-inoculation (pi) onwards. Viruses *wt*Dl965 and *r*Dl965 showed similar replication curves, except at 2 dpi (Fig. 1), when *r*Dl965 titre was significantly lower than *wt*Dl965 titre (p = 0.0001). Maximum mean titres for *wt*Dl965, *r*Dl965 and Mut247Dl965 were recorded at 5 dpi (1.9 × 10^5^, 5.5 × 10^5^, and 3.8 × 10^5^ TCID_50_/ml, respectively), whereas Mut270Dl965 and Mut247 + 270Dl965 maximum titres were at 7 dpi, with mean values of 7.1 × 10^5^ and 1 × 10^5^ TCID_50_/ml, respectively. The double mutated virus (Mut247 + 270Dl965) showed the most important significant differences compared to *wt*Dl965 (p < 0.0001). Thus, at 2 and 5 dpi, Mut247 + 270Dl965 titres were 1.3 × 10^3^ and 3.9 × 10^4^ TCID_50_/ml, respectively, values significantly lower (p < 0.0001) than those obtained for *wt*Dl965 (1.3 × 10^4^ and 1.9 × 10^5^ TCID_50_/ml, respectively).

### Comparative analysis of *wt*Dl965 and *r*Dl965 virulence

**Figure 2:**
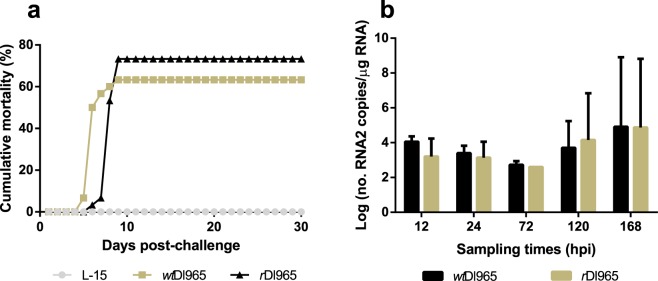
Comparative analysis of *wt*Dl965 and *r*Dl965 virulence. (**a**) Accumulated mortality. (**b**) Viral genome quantification in European sea bass brain. Viral genome was quantified by RT absolute real-time PCR. The t-student test was used to compare the number of RNA2 copies in fish from different experimental groups within each sampling time. Values of p < 0.05 were considered significant. Results are mean ± s.d. (n = 3).

Typical signs of disease and mortality were recorded in both infected groups. First disease signs, consisting of dark skin and loss of appetite, were recorded at 3 dpi, whereas typical neurological signs, such as abnormal swimming, appeared at 5 dpi. The accumulated mortality at the end of the experiment was 63.3 and 73.3% for *wt*Dl965- and *r*Dl965-infected groups, respectively (Fig. 2a). The statistical comparison between survival distributions (Kaplan-Meyer survival curve and log-rank Mantel Cox analysis) showed no significant differences (p = 0.369) between both groups (Supplementary Fig. S1). No mortalities were recorded in the negative control group (L-15-exposed fish).

In fish infected with *wt*Dl965, mortality onset was at 5 dpi, and an acute episode of mortality was observed at 6 dpi, increasing the accumulated mortality from 6% to 50% in just one day. Fish mortality stabilized at 10 dpi (Fig. 2a). In *r*Dl965-infected group, first mortalities were recorded at 6 dpi, with the maximum daily mortality at day 8 pi. After this episode, mortality stabilized at 10 dpi (Fig. 2a). In order to analyze the viral replication, the number of RNA2 copies was quantified in brains sampled at different times pi. Viral RNA was detected in sea bass infected with both viruses at all sampling times analysed, whereas no viral genome was recorded in the negative control group (Fig. 2b). In addition, no significant differences (p > 0.05) between both infected groups were recorded at any time, indicating that the pathogenesis of both viruses, in terms of replication and migration to brain, is similar.

### Role of Cp amino acids 247 and 270 in betanodavirus virulence

In this challenge, *r*Dl965 was used as positive control, since it shows the same virulence as *wt*Dl965 (Fig. 2a), and it was generated by reverse genetics, as the mutated viruses.

**Figure 3:**
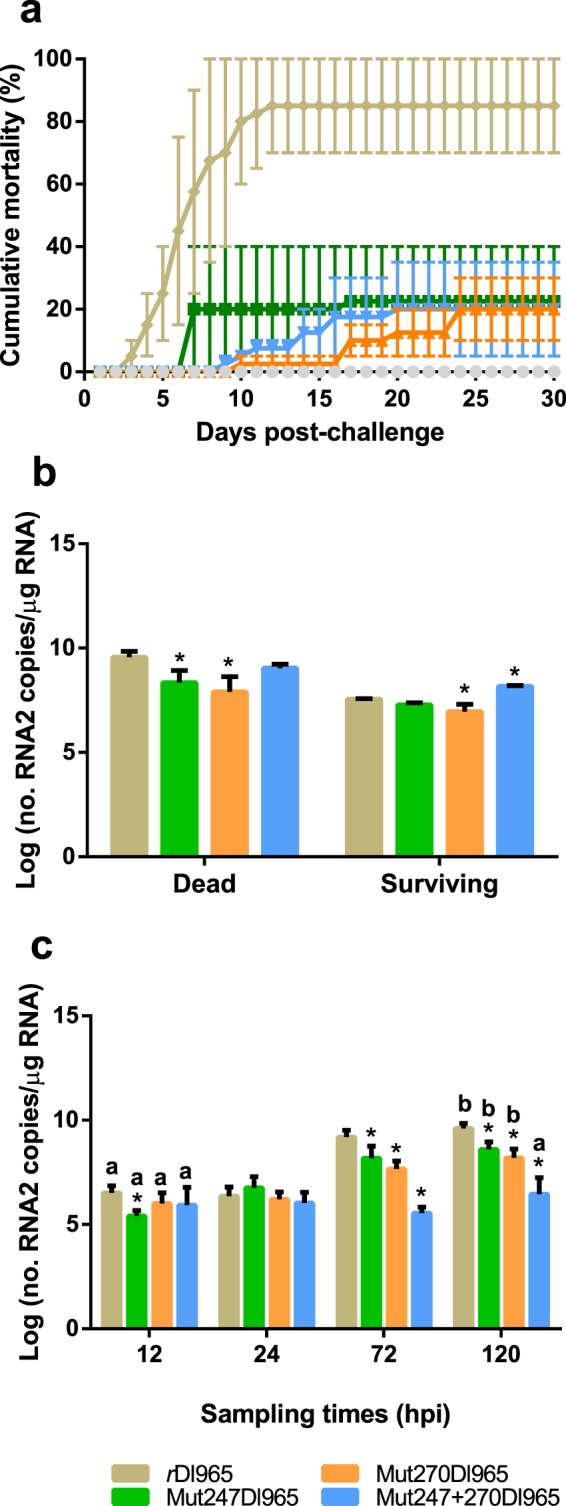
Virulence analysis of *r*Dl965 and mutated viruses. (**a**) European sea bass mortality inoculated with different recombinant viruses. (**b**) Viral RNA2 quantification in brain from dead and surviving fish. The analysis was performed by RT absolute real-time PCR. The one-way ANOVA was the statistical analysis conducted. Asterisk indicates significant differences, compared to the *r*Dl965 group, within each group of animals (dead or surviving) (p < 0.05). Results are mean ± s.d. of two pools of three brains. (**c**) RNA2 quantification in brain from sampled sea bass. The one-way ANOVA and t-student tests were the statistical analyses performed. Asterisk indicates significant differences between each mutated virus and *r*Dl965 within each sampling time (p < 0.05). Different letters indicate significant differences between 12 h and 5 dpi within each experimental group (p < 0.05). Results are mean ± s.d. (n = 5).

In *r*Dl965-infected group, typical signs of disease (dark pigmentation, loss of appetite and abnormal swimming) were observed from 3 to 12 dpi, with a peak of daily mortality at day 7 pi. After this period, mortality stabilized (85% at the end of the challenge, 30 dpi) and animals recovered (Fig. 3a). Fish infected with Mut247Dl965 also showed a mortality peak at day 7 pi, and the accumulated mortality at the end of the experiment was only 22.5% (Fig. 3a). In Mut270Dl965-infected group, disease signs appeared later and were less severe than in fish infected with *r*Dl965. The final accumulated mortality in this group was 20%. In fish infected with Mut247 + 270Dl965, signs appearance and mortality onset were also delayed (9 dpi), and accumulated mortality was 20% (Fig. 3a). The log-rank Mantel Cox analysis showed significant differences (p < 0.0001) in the survival distribution estimated for fish infected with mutant strains compared to that of the *r*Dl965-infected group (Supplementary Fig. S2). However, the survival distribution in fish challenged with the three mutated viruses was statistically similar (Supplementary Fig. S2).

Viral genome was detected in brains from both, dead and surviving fish, obtained from all the infected groups. The RNA2 copy number in dead animals from Mut247Dl965- and Mut270Dl965-infected groups was significantly lower (p < 0.05) (3.2 × 10^8^ and 1.4 × 10^8^ RNA2 copies/μg RNA, respectively) than in dead fish from the *r*Dl965-infected group, showing 4.1 × 10^9^ RNA2 copies/μg RNA (Fig. 3b).

Regarding surviving animals, the mean number of RNA2 copies was significantly lower (p < 0.0001) in the Mut270Dl965-infected group (1 × 10^7^ RNA2 copies/μg RNA) than in the *r*Dl965-infected group (3.6 × 10^7^). On the contrary, brains from surviving sea bass infected with Mut247 + 270Dl965 displayed a significantly higher RNA2 copy number (1.5 × 10^8^ RNA2 copies/μg RNA) than samples from the *r*Dl965-infected group (p < 0.0001) (Fig. 3b).

In order to complete this study, brains from fish sampled at different time points were analysed. Viral genome was not recorded in fish from the negative control group, whereas all recombinant viruses were detected at all sampling times considered (Fig. 3c). At early stage of infection (12 and 24 hpi), the number of RNA2 copies was similar in samples from all the experimental groups, except for the Mut247Dl965-inoculated group at 12 hpi. However, at 3 and 5 dpi, fish infected with mutant viruses showed RNA2 values significantly lower than those recorded in fish from the *r*Dl965-infected group (p < 0.0001). In addition, the number of *r*Dl965, Mut247Dl965 and Mut270Dl965 RNA2 copies increased throughout the time, with maximum mean values at 5 dpi (4.4 × 10^9^, 4.8 × 10^8^, and 2.3 × 10^8^ RNA2 copies/μg RNA, respectively). In contrast, a significant increase of Mut247 + 270Dl965 RNA2 copy number was not recorded up to 5 dpi (p > 0.05) (Fig. 3c).

### Transcription of immunogenes

**Figure 4:**
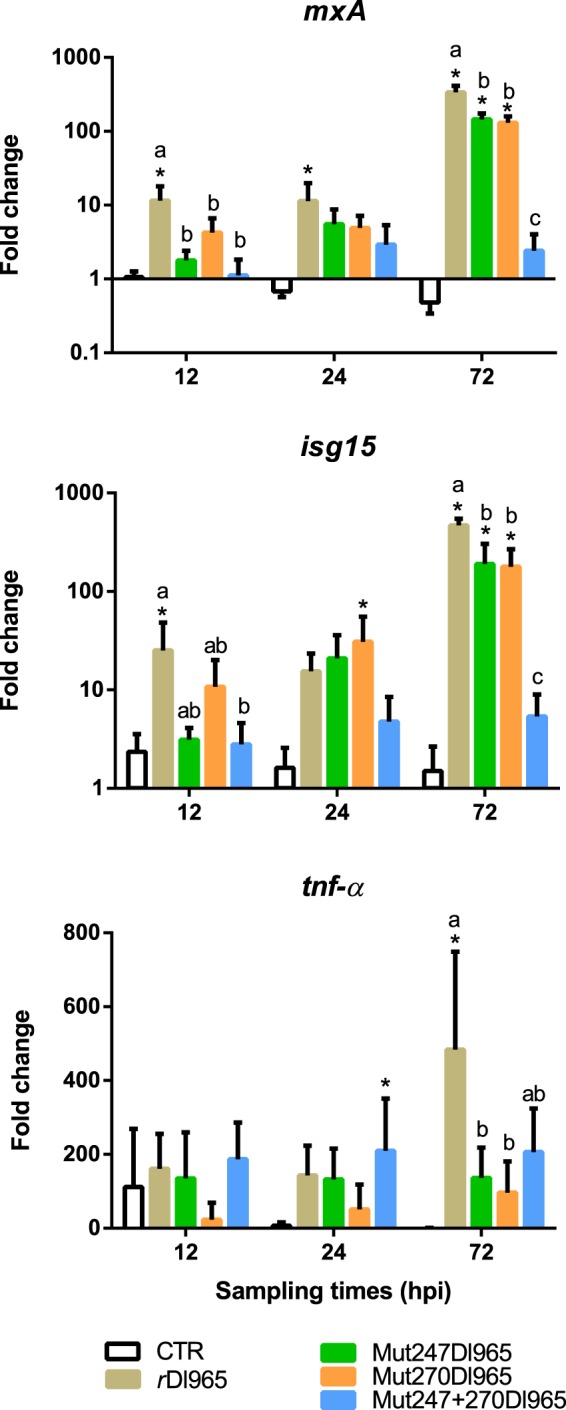
Relative quantification of *mxA*, *isg15* and *tnf-α* transcription after inoculation with *r*Dl965 or mutated viruses. Data were statistically analysed with the one-way ANOVA test. Asterisk indicates significant differences between L-15-injected (negative control) and virus-inoculated fish within each sampling time (p < 0.05). Different letters denote significant differences among experimental groups at the same sampling time (p < 0.05). Results are mean ± s.d. (n = 5).

The innate immune response after infections with the recombinant viruses was analysed by quantification of *mxA*, *isg15* and *tnf-alpha* transcription. Only *r*Dl965 induced significantly *mxA* and *isg15* transcription at the first sampling time considered (12 hpi) (Fig. 4), with mean fold change values of 12.5 (p = 0.0002) and 25.2 (p = 0.0174), respectively. The transcription of both genes was maximal in fish infected with this virus at 72 hpi, with mean fold change values of 336 and 470, for *mxA* and *isg15*, respectively. These values were significantly higher (p < 0.0001) than those recorded at the same sampling time in brains from Mut247Dl965- and Mut270Dl965-infected fish (Fig. 4). In addition, Mut247 + 270Dl965 did no trigger the transcription of these ISGs at any time. Concerning the induction of the pro-inflammatory cytokine *tnf-alpha*, significant values of transcription were only recorded after *r*Dl965 inoculation at 72 hpi (483.5 mean fold change value) (p = 0.0004) and in Mut247 + 270Dl965-inoculated fish sampled at 24 hpi, with a mean fold change value of 206 (Fig. 4) (p = 0.0121).

### Analysis of anti-betanodavirus antibodies in sera

**Figure 5:**
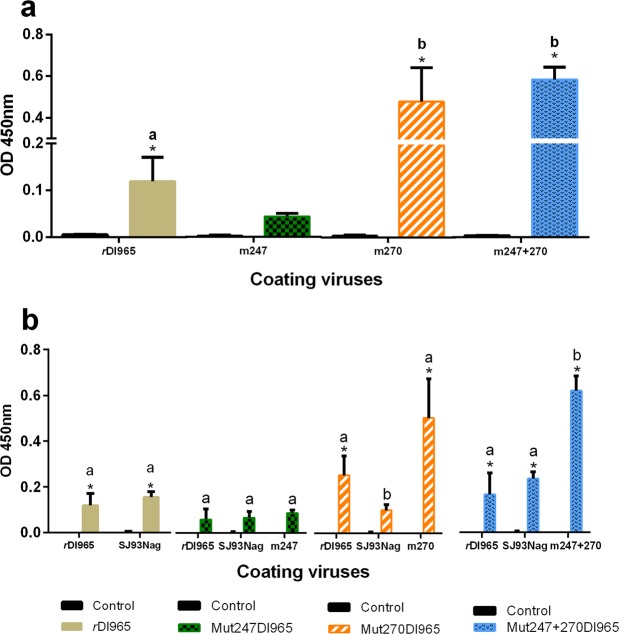
Serological study of sea bass infected with recombinant viruses. (**a**) Level of anti-betanodavirus antibodies evaluated by indirect ELISA. Different letters denote significant differences among infected groups (p < 0.05). (**b**) Cross-reaction between antibodies in sera from European sea bass inoculated with *r*Dl965, Mut247Dl965, Mut270Dl965 or Mut247 + 270Dl965, and different viral antigens (lysates of E-11 cells inoculated with the different viruses considered). Different letters denote significant differences among different coating viruses within each experimental group (p < 0.05). Data were statistically analysed with the one-way ANOVA test. Asterisk indicates significant differences (p < 0.05) between control (sera from L-15-injected fish) and infected groups. Results are mean ± s.d. of three samples composed of sera from five fish. All sera were analysed at 1/64 dilution.

As it is shown in Fig. 5a, a significant production of anti-betanodavirus antibodies was recorded in *r*Dl965-, Mut270Dl965- and Mut247 + 270Dl965-infected fish compared to the negative control group (L-15-injected group) (p < 0.0001). However, a measurable level of antibodies was not recorded in sera from Mut247Dl965-inoculated fish. In addition, antibody production was significantly higher in animals infected with the recombinant viruses Mut270Dl965 and Mut247 + 270Dl965 compared to antibodies in fish from the *r*Dl965-infected group (Fig. 5a) (p < 0.0001).

To evaluate possible changes in NNV serological properties caused by amino acid mutations at positions 247 and 270, a cross-reaction assay was performed (Fig. 5b). Antibodies in sera from *r*Dl965- and Mut247 + 270Dl965-infected fish recognized RGNNV (*r*Dl965 strain) and SJNNV antigens (SJ93Nag, reference strain). Maximum mean OD values were obtained for fish in the Mut247 + 270Dl965 group (0.15, 0.22, and 0.58 for RGNNV, SJNNV and Mut247 + 270Dl965 coating virus, respectively). However, antibodies in sera from fish infected with Mut270Dl965 did not cross-react with SJNNV antigens, recognizing only Mut270Dl965 as coating virus (0.52 mean OD value), used as positive control, and *r*Dl965 antigens (0.28 mean OD value) (Fig. 5b).

## Discussion

In the present study, the role of the Cp amino acids 247 and 270 as betanodavirus virulence determinants to European sea bass has been evaluated. With this aim, recombinant viruses displaying mutations in the mentioned positions were obtained by reverse genetics. This technique has been successfully used to study both, negative- and positive-stranded RNA fish viruses^11,19–25^.

The *r*Dl965 virus, which showed *in vitro* replication and *in vivo* virulence similar to the wild type virus (*wt*Dl965), was successfully recovered by reverse genetics. Hereupon, amino acids at positions 247 (serine) and 270 (serine) were substituted by those present in SJNNV isolates (alanine and asparagine, respectively), which are low virulent to European sea bass^7,11,16,17^. The effect of these modifications was first analysed by evaluating viral replication kinetics on E-11 cells (Fig. 1), with the double mutant virus showing the most important significant differences compared to *wt*Dl965 replication (p < 0.0001). This result is in contrast with that previously reported for recombinant reassortant strains (RGNNV/SJNNV) with substitutions at the same positions^11^. Since strains belonging to different genotypes were used in both assays, it seems that modification of both amino acids in a RGNNV capsid could have a more important effect than in a SJNNV capsid. Our findings suggest that the double mutation may affect viral entry by altering recognition of the host receptor, which in E-11 cells is an acid sialic^26,27^. Further studies analysing virus and E-11 interaction would be necessary to confirm this hypothesis.

The mortality caused by *r*Dl965 after intramuscular injection was 85%, which is similar to mortality rates previously reported for RGNNV isolates, ranging between 36% and 75%, depending on the infection route, temperature, salinity, viral isolate or viral dose^16–18,28,29^. However, mortality caused by mutated viruses was significantly lower (22.5%, 20%, and 20%, for fish infected with Mut247Dl965, Mut270Dl965, or Mut247 + 270Dl965, respectively). These rates are similar to those reported in sea bass inoculated with SJNNV isolates, which ranged between 10%^16^ and 0%^17,18^. In addition, the course of disease in these infected groups was altered, and disease signs were weaker in intensity and duration, indicating that amino acids 247 and 270 may play an important role as virulence determinants in sea bass, similar to that previously reported in Senegalese sole^11^. However, a virus causing no mortality was not obtained, which suggests the involvement of additional Cp amino acids in betanodavirus virulence to European sea bass. In addition, the double mutation did not result in a lower mortality rate compare to single-mutated viruses, which agrees with previous results reported in Senegalese sole after infection with a double-mutated reassortant virus^11^. However, the same double-mutated virus caused lower mortality than single-mutated viruses in turbot (*Scophthalmus maximus*)^30^. These results suggest the involvement of unknown host factors (probably different cellular receptor) and support the importance of these amino acids also as host determinants.

The number of RNA2 copies in dead sea bass infected with single mutated viruses was lower than in fish infected with *r*Dl965, which could be related to the low mortality caused by the mutant viruses. Viral genome was detected in all surviving fish analysed, although the number of RNA copies was lower than that observed in dead animals, as it has been previously reported^18^. This result may suggest the establishment of an asymptomatic infection, and the role of this fish as asymptomatic carrier. However, additional experiments would be necessary to test fish at long term and to check if animals would eventually eliminate the virus or establish a non-productive infection. Comparative analyses of RNA2 copy number in sampled animals revealed that amino acid modifications affect viral replication in sea bass brain, which is coherent with the lower mortality caused by the mutant viruses (Fig. 3c). Furthermore, the number of RNA2 copies of the virus harbouring double mutation was significantly lower than *r*Dl965 and single-mutant virus genome copy number at 5 dpi. In addition, the *in vivo* replication of the double mutant virus is similar to that observed *in vitro*, with a lagged replication on E-11 cells compared to the non-mutated virus replication. These variations could be related to alterations in cell receptor binding. As it has been previously reported, a single modification in the influenza virus hemagglutinin receptor-binding domain changes the cell-receptor recognition^31^. Furthermore, since these amino acids are located within the P-domain^10^, their alteration may affect the physicochemical properties of the capsid, including cell-receptor recognition.

Important differences between the immune response induced by mutated viruses and by *r*Dl965 were observed, being the first one similar to that described after SJNNV inoculation^32^. The results of transcription of *mxA* and *isg15* genes showed that *r*Dl965 is a strong inductor of sea bass IFN I system in brain, whereas single mutants prompted lower levels of transcription, which is coherent with the lower number of RNA2 copies recorded in infected fish. Maximum levels of transcription were always at 72 hpi, as it has been previously reported^33–35^. Moreover, no induction of these genes was detected in animals infected with the double mutant, in which no significant viral replication was recorded.

The importance of inflammatory processes in betanodavirus disease has been previously described^33,36,37^. Our results showed a strong induction of this gene in brain from *r*Dl965-inoculated fish at 72 hpi, as it has been previously reported^33^, whereas low or no induction was observed in fish infected with less virulent mutant strains. These findings and the delayed and weaker disease sign appearance in fish infected with the mutant strains, suggest that the uncontrolled up-regulation of this pro-inflammatory cytokine could be partially responsible for the histological damages in brain, retina and spinal cord observed in infected fish, as it has been proposed by Labella *et al*.^38^ based on a RNAseq study on betanodavirus pathogenesis in Senegalese sole.

Enzyme-Linked ImmunoSorbent-Assay (ELISA) analyses showed anti-NNV antibodies in sera from all infected groups, except for fish inoculated with Mut247Dl965, which did not trigger an antibody production measurable by the ELISA procedure used in this study (analysis of 1/64-diluted sera). In fish inoculated with the other mutant strains, antibody production was significantly higher than in animals from *r*Dl965 group (p < 0.0001). Similarly, a previous study has reported that a SJNNV isolate, with low virulence to sea bass, triggered a higher level of antibodies than a highly virulent RGNNV isolate^32^, which partially agrees with the results obtained in this study. Both findings suggest an inverse relationship between virulence and antibody production. To further evaluate the serological changes in mutated viruses, a cross-reaction assay was conducted by indirect ELISA. Antibodies in fish infected with *r*Dl965 or with the double mutant recognized RGNNV and SJNNV antigens at similar level, which has been previously reported using other RGNNV isolates^39^, whereas antibodies in sera from fish infected with Mut270Dl965 recognized only RGNNV antigens. Therefore, mutation in amino acid 270 within the RGNNV Cp sequence seems to modify importantly the serological properties of the *r*Dl965 virus. A more extensive study, including a wide range of RGNNV and SJNNV isolates and the titre of neutralizing antibodies, seems to be necessary to fully understand the implications of these variations in antigen-antibody cross-reactivity and protection. In fact, a recent study has reported high seroconversion in sea bass vaccinated with inactivated SJNNV, although with low neutralization properties against RGNNV infections, which indicated lack of *in vivo* cross-protection^39^. Noteworthy, the double mutation resulted in the generation of a virus able to induce the highest seroconversion, and antibodies in sera from these animals recognized both, RGNNV and SJNNV antigens. For these reasons, this virus may be a valuable potential candidate for anti-betanodavirus vaccine development, although a more extensive study on the reactivity and neutralizing properties of antibodies should be performed.

In conclusion, this study has demonstrated the importance of capsid amino acids 247 and 270 as virulence determinants in betanodavirus infection in sea bass. Substitution of these amino acids to those present in an SJNNV-type Cp caused a significant decrease in viral virulence. Furthermore, mutant viruses triggered a reduced transcription of *mxA*, *isg15* and *tnf-alpha* genes, inducing, however, higher production of antibodies (except for Mut247Dl965). In addition, the double mutant elicited the highest level of antibodies, being able to recognize both, RGNNV and SJNNV antigens, and, for this reason, it could represent a first step in betanodavirus vaccine development.

## Methods

### Viral isolate, titration and cell culture

The SpDl_IAusc965.09 isolate (RGNNV), obtained from diseased European sea bass in the Aquaculture Institute of Santiago de Compostela (Spain), has been used in this study. This isolate will be named as *wt*Dl965 to establish a clear differentiation with the isolate generated by reverse genetics (*r*Dl965).

Viruses were propagated on the E-11 cell line^27^ using Leibovitz L-15 medium supplemented with 2% fetal bovine serum (FBS), 100 IU/ml penicillin and 10 mg/ml streptomycin at 25 °C until fully CPE development. The resulting viral suspensions were titrated following the 50% tissue culture infective dose method (TCID_50_)^40^. These viral stocks were stored at −80 °C until used.

BSRT7/5 cells^41^, kindly provided by Dr K.K. Conzelmann (Ludwig-Maximilians-Universität Munich, Germany), were used to generate infective viral particles by reverse genetics. These cells were grown using Dulbecco’s Modified Eagle Medium (DMEM) supplemented with 10% FBS, 200 mM glutamine, 100 IU/ml penicillin, and 10 mg/ml streptomycin. Cells were incubated at 37 °C in a 5% CO_2_ humidified atmosphere, adding 1 mg/ml geneticin (G418) every other sub-culture.

### Sequencing of RNA1 and RNA2 genomic segments

A two-step strategy has been followed to obtain the complete *wt*Dl965 sequence from viral suspensions: (i) sequencing of RNA1 and RNA2 open reading frames (ORFs), using the primer walking approach^5^; (ii) sequencing of 5′- and 3′-ends by the Rapid Amplification of cDNA Ends (RACE) methodology^11^.

### Recovery of *r*Dl965 by reverse genetics

Total RNA was extracted from semi-purified *wt*Dl965 viral particles, using the TRI reagent solution, and subsequently reverse-transcribed using the SuperScript™ III Reverse Transcriptase (Invitrogen). Primers used to amplify the complete cDNA sequences were T7_5′RNA1_965 and 3′RNA1_965, for RNA1 amplification, and T7_5′RNA2_965 and 3′RNA2_965, to amplify the RNA2 segment (Supplementary Table S1). These primers contain several sequences required for cloning and reverse genetics. Thus, forward primers (T7_5′RNA1_965 and T7_5′RNA2_965) include *BamHI* and *SacII* restriction sites, the T7 polymerase promoter sequence, and two guanine residues^19,42^. Reverse primers (3′RNA1_965 and 3′ RNA2_965) display a blunt-end *SfoI* restriction site (Supplementary Table S1).

Amplifications were performed in 50-μl mixtures composed of 1x Pfx Amplification Buffer, 0.3 mM dNTPs, 1 mM MgSO_4_, 0.3 µM specific primers (Supplementary Table S1), Platinum™ Pfx DNA Polymerase (1 U, Invitrogen) and cDNA (200 ng). The amplification profile was: denaturation at 95 °C for 5 min, followed by 35 cycles of amplification at 95 °C for 1 min, 60 °C for 30 s, 72 °C for 1.5 min, and a final elongation at 72 °C for 10 min. Amplified products were purified using the Illustra GFX PCR DNA and Gel Band Purification Kit (GE Healthcare), quantified using the NanoDrop-1000 system, and finally cloned in the pGEM®-T Easy Vector (RNA1, Promega) or CloneJET Vector (RNA2, Fermentas) according to the standard procedure. *Escherichia coli* DH5α cells were transformed by electroporation, and selected clones were confirmed by sequencing.

Constructions harbouring the RNA1 or RNA2 segment (pGemT_BamHI_RNA1 or pJET_RNA2, respectively), were subcloned into the eukaryotic expression plasmid *pBSδRiboT7t*^22^, which was kindly supplied by Dr M. Bremont (Unité de Virologie et Immunologie Moléculaires, INRA, Jouy en Josas, France) and used to transform *E. coli* DH5α cells. Plasmids (labelled as *pBSRNA1* and *pBSRNA2*) were purified with the Genopure Plasmid Maxi Kit (Roche) according to manufacturer instructions. The RNA1 or RNA2 insert presence was checked by using the NNVs1_B3F/NNVs1_B3R or NNVs2_RG2F/NNVs2_RG2R pairs of primers (Supplementary Table S1), respectively.

### Generation of recombinant mutated viruses

The construction *pBSRNA2* was used as template to mutate amino acids 247 and 270 within the viral Cp sequence. Specifically, Mut247Dl965 primer (5′-GTCCATCCTCCTAGGAGCCACACCACTGGAC-3′) introduces a mutation affecting nucleotide 759 (thymine to guanine), which switches serine in position 247 to alanine. The change generated by Mut270Dl965 primer (5′-TCCGCTGTCTATTGACTACAACCTTGGAACTGGAG-3′) occurs in the nucleotide 830 of the RNA2 sequence (guanine to adenine), changing the amino acid in position 270 (serine to asparagine). These modifications were carried out using 50 ng of DNA and the QuikChange Multi Site-Directed Mutagenesis Kit (Agilent Technologies) according to manufacturer’s instructions. Mutations were confirmed by PCR, using T7_5′RNA2_965/3′RNA2_965 primers (Supplementary Table S1), and the subsequent sequencing of the amplified products.

Three different constructions, named as Mut247pBSRNA2, Mut270pBSRNA2 and Mut247 + 270pBSRNA2, were obtained following this procedure.

### Transfection of BSRT7/5 cells and recovery of infective viral particles

The plasmid *pBSRNA1*, in combination with *pBSRNA2*, Mut247pBSRNA2, Mut270pBSRNA2 or Mut247 + 270pBSRNA2, were used to transfect BSRT7/5 cells with the Lipofectamine® 2000 Transfection Reagent (Invitrogen), as previously described^11^. The resulting viral suspensions were diluted in L-15 medium (1/10, 1/100 and 1/1000) to be inoculated on E-11 cells grown on 24-well plates. These inoculated cells were cultured in L-15 medium supplemented with 2% FBS, 100 IU/ml penicillin and 10 mg/ml streptomycin at 25 °C for 7 d. When it was required, blind passages were performed once a week until the development of CPE. Viral particles obtained (*r*Dl965, Mut247Dl965, Mut270Dl965, and Mut247 + 270Dl965) were propagated and titrated on E-11 cells, and stored at −80 °C until used. The mutations were demonstrated by sequencing.

### *In vitro* viral replication

Each virus was inoculated at 0.1 multiplicity of infection (MOI) on E-11 cells grown on 24-well plates. Inoculated cells were maintained in L-15 medium supplemented with 100 IU/ml penicillin and 10 mg/ml streptomycin at 25 °C for 1 h for virus adsorption. After this incubation, viral suspensions were removed, and L-15 medium supplemented with 2% FBS, 100 IU/ml penicillin and 10 mg/ml streptomycin was added. Supernatants from three wells were collected at 1, 2, 3, 5, and 7 dpi to be titrated on E-11 cells by the TCID_50_ method. All titrations were conducted in triplicate.

### *In vivo* viral virulence analysis. Experimental challenges and sample processing

#### *wt*Dl965 *versus r*Dl965

Juvenile sea bass specimens (6 g, average weight) were distributed into 6 tanks in order to establish 3 experimental groups in duplicate (tanks A and B) (n = 30 each): (i) *wt*Dl965-infected group, (ii) *r*Dl965-infected group, and (iii) L-15-treated group, as negative control. Infections were performed by 1-h immersion in seawater containing a final viral concentration of 10^5^ TCID_50_/ml. Fish from tanks A were daily monitored, for 30 days, to record clinical signs and mortality. Fish from tanks B were sampled at 12 hpi, as well as at 1, 3, 5, and 7 dpi (3 specimens per time point). Their brains were aseptically collected and maintained at −80 °C until RNA2 quantification by real-time PCR. Water temperature was always maintained at 25 °C. Sampled and surviving fish were killed by MS-222 (Sigma) anaesthetic overdose.

#### *r*Dl965 *versus* mutant viruses

In order to analyse possible virulence changes resulting from amino acid modifications, fish (8 g, average weight) were intramuscularly infected (2 × 10^6^ TCID_50_/fish) with *r*Dl965, Mut247Dl965, Mut270Dl965, or Mut247 + 270Dl965 (n = 25 each) in triplicate. In addition, a control group, injected with L-15, was set up. Water temperature was maintained at 25 °C throughout the experiment. Fish from two of the triplicate tanks were daily monitored for 30 d to record mortality, and specimens from the remaining tanks were sampled at 12 hpi, as well as at 1, 3, and 5 dpi. Five animals were killed per sampling time and group by MS-22 overdose, and their brains were individually collected and stored in liquid nitrogen for subsequent virological and immunological analyses. In addition, brains from dead and surviving fish were pooled (two pools of three brains) and stored until virological analysis. Finally, 15 surviving fish per group were anesthetized for blood extraction from the caudal vein at 30 dpi. Three samples, composed of blood from 5 specimens, were used to perform antibody study. Sera were obtained by overnight incubation at 4 °C for clotting, followed by two centrifugations at 400 *xg* for 15 min at 4 °C. The resulting sera were stored at −20 °C until used.

Brains were homogenized (10%, w/v) in TRI reagent solution to extract total RNA. After treatment with DNase I Recombinant from bovine pancreas, cDNA was synthesized using 1 μg of RNA and the Transcriptor First Strand cDNA Synthesis Kit according to manufacturer’s guidelines. cDNA was quantified with the NanoDrop-1000 system and maintained at −20 °C until used.

### Viral genome quantification

RNA2 segment was quantified by absolute real-time PCR using the specific primers RG_965_RNA2 F4 and RG_965_RNA2 R1 (Supplementary Table S1). Serial dilutions of the *pJET* vector containing the complete *wt*Dl965 RNA2 sequence were used to generate reference standard curves. All the amplifications were conducted with the LightCycler 96 Thermocycler as previously described^35^.

### Transcription of immunogenes

The transcription of *mxA*, *isg15* and *tnf-alpha* genes has been quantified by relative real-time PCR using ribosomal 18S RNA as reference endogenous gene (Supplementary Table S1). PCRs were performed with the LightCycler 96 Thermocycler in 20-µl mixtures composed of cDNA generated from 50 ng of RNA, 1x Fast Start Essential DNA Green Master and 10 pmol specific primers (Supplementary Table S1). Amplification consisted of 95 °C for 10 min, followed by 45 cycles at 95 °C for 10 s, 52 °C for 10 s and 72 °C for 10 s. Melting curves were obtained at 95 °C for 10 s, 65 °C for 60 s and 97 °C for 1 s. Relative fold change values were calculated using the Pfaffl method^43^.

### Indirect ELISA for anti-betanodavirus antibody analyses

The level of anti-betanodavirus antibodies in sera from experimentally-infected sea bass, as well as the possible changes in viral serological properties caused by the mutations included in the Cp sequence, have been evaluated following the indirect ELISA procedure previously reported^44^. Optical density (OD) was measured at 450 nm using the ELISA Whittaker Microplate Reader 2001. Sera obtained from sea bass infected with the different recombinant viruses were analysed using a 1/64 dilution in PBS. Resulting OD values were normalized by subtracting the OD values of the negative control (omitting fish sera) wells.

### Statistical analyses

Results were statistically analysed with the GraphPad Prism 6 software. Values of p < 0.05 were considered significant. Mortality rates were analysed by the survival curves, using the Kaplan-Meyer test. To determine significant differences in survival distributions, a log-rank Mantel Cox test was carried out. Viral quantification results from the “*wt*Dl965 *versus r*Dl965” challenge were analysed by the t-student test, whereas all the results derived from the “*r*Dl965 *versus* mutated viruses” challenge were analysed by the one-way ANOVA test, using Bonferroni’s multiple comparison test as post-test. Normality distribution was verified by Shapiro-Wilk test.

### Ethic statement

All *in vivo* procedures were always performed according to the European Union guidelines for the handling of laboratory animals (Directive 2010/63/UE). The lowest stress-generating conditions of light (8-h light and 16-h darkness photoperiod), oxygen (6 ± 0.5 ppm) and feeding were applied throughout all the experimental challenges. All procedures were authorized by the Bioethics and Animal Welfare Committees of Universities of Malaga and Santiago de Compostela, and given the registration numbers CEUMA 52-2017-A and 15004/13/002 by the National and Galician authorities for regulation of animal care and experimentation, respectively.

## Supplementary information

Supplementary Figures S1 and S2, and supplementary Table S1
