# Supplementary material for: Capsid amino acids at positions 247 and 270 are involved in the virulence of betanodaviruses to European sea bass
Source: Sci Rep. 2019 Oct 1;9:14068. doi: 10.1038/s41598-019-50622-1 (PMC6773868; doi:10.1038/s41598-019-50622-1)
Supplement: Supplementary file 1 — Supplementary Figures S1 and S2, and supplementary Table S1 [file 41598_2019_50622_MOESM1_ESM.pdf]

Capsid amino acids at positions 247 and 270 are involved in the virulence of betanodaviruses to European sea bass

Patricia Moreno, Sandra Souto, Rocio Leiva-Rebollo, Juan J. Borrego, Isabel Bandín & M. Carmen Alonso*

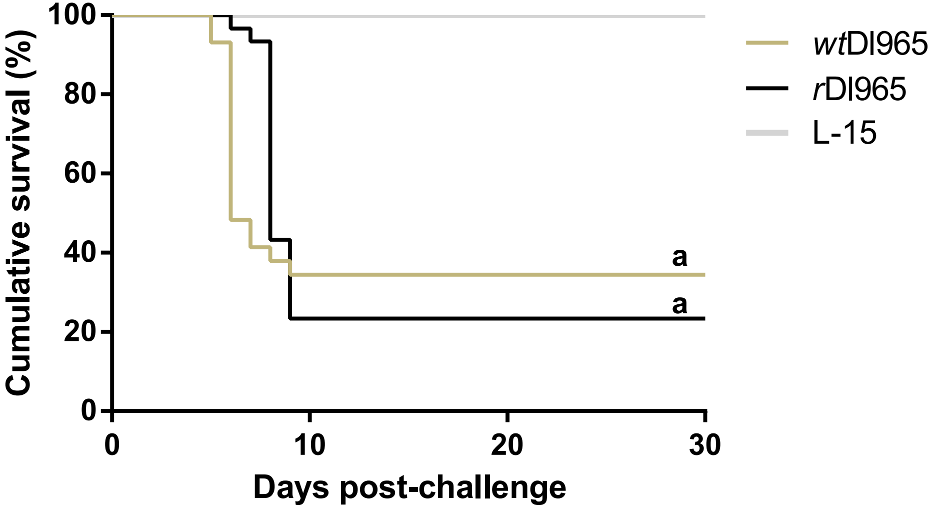

	Chi square	Df	p value
Log Rank Mantel Cox	0.857	1	0.369

Figure S1. Comparative analysis between *wt*DI965 and *r*DI965 virulence: Kaplan-Meyer survival curves. Log Rank Mantel Cox Test results are represented below the Kaplan-Meyer survival curve. Same letters indicate no significant differences ($p < 0.05$).

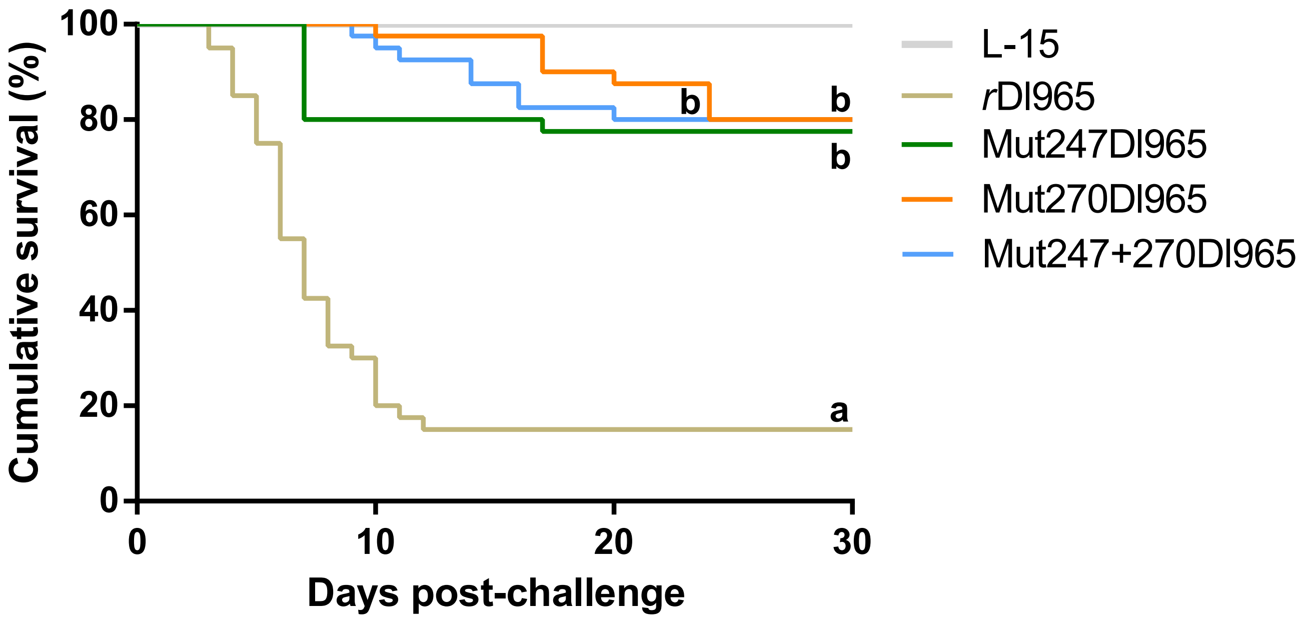

Log Rank Mantel Cox	Chi square	Df	p value
*r*DL965 vs Mut247DI965	36.47	1	< 0.0001
*r*DL965 vs Mut270DI965	52.86	1	< 0.0001
*r*DL965 vs Mut247+270DI965	48.55	1	< 0.0001
Mut247 vs Mut270 vs Mut247+270	0.2822	2	0.8684

Figure S2. Comparative analysis between *r*DI965 and mutated virus virulence: Kaplan-Meyer survival curves. The log Rank Mantel Cox Test results are in a box below the Kaplan-Meyer curves. Different letters indicate significant differences between experimental groups ($p < 0.05$).

Table S1.- Primers used in this study. (a) Primers designed to amplify the full-length RNA1 and RNA2 segments. Several motifs are represented in colour: *BamHI* cut site in grey, *SacII* cut site in green, the T7 promoter sequence in blue, two guanine residues in red and *SfoI* blunt-ended cut site in orange. (b) Set of primers used to confirm the presence of viral RNA1 and RNA2 segment during the clonation procedure. (c) Primers used for absolute quantification of RGNNV RNA2 viral segment. (d) Primers used for relative quantification of immunogene transcription.

Name	Sequence (5'-3')	Product length (bp)	References
**a**			
**T7_5'RNA1_965**	GGATCCCCGCGGTAATACGACTCACTATAGGTAACATCCCTTTCTTGCTCTGTT	3,104	This study
**3'RNA1_965**	GGCGCCGAAGCGTAGGACAGCATAAAGC		
**T7_5'RNA2_965**	GGATCCCCGCGGTAATACGACTCACTATAGGTAATCCATCACCGCTTTGCAATC	1,432	This study
**3'RNA2_965**	GGCGCCGAGTTGAAAAGCGATCAGCGG		
**b**			
**NNVs1_B3F**	AACATCCGCACTGCATACGAACTG	615	Olveira et al., (2009)[5]
**NNVs1_B3R**	ATGCTGGAGAACACTGGCTTTGAA		
**NNVs2_RG2F**	CTTCCTGCCTGATCCAACTGACAACG	568	Olveira et al., (2009)[5]
**NNVs2_RG2R**	CCAGATGCCCCAGCGAAACCA		
**c**			
**RG_965_RNA2 F4**	ACCGTCCGCTGTCTATTGACTA	126	Moreno et al., (2016)[35]
**RG_965_RNA2 R1**	CAGATGCCCCAGCGAAACC		
**d**			
**rRNA18S Fw**	CCAACGAGCTGCTGACC	208	Scapigliati et al., (2010)[45]
**rRNA18S Rw**	CCGTTACCCGTGGTCC		
**Dl_MxA Fw**	ATTCTGAGTTCTTGCTGAAGG	113	Novel et al., (2013)[34]
**Dl_MxA Rw**	CCTCTAGAACTCCACCAGG		
**qISG15 F2**	CGACTCAAAGCCTCTCTGCTACT	100	Moreno et al., (2016)[35]
**qISG15 R2**	CGTTTCTGACGAACACCTGGAT		
**Dl_TNFα Fw**	CGACTGGCGAACAACC	220	Nascimento et al., (2007)[46]
**Dl_TNFα Rw**	GCTGTCCTCCTGAGC		
